# Three misdiagnoses before a final diagnosis of 17α-hydroxylase/17,20-lyase deficiency: A case report

**DOI:** 10.1097/MD.0000000000043467

**Published:** 2025-07-25

**Authors:** Jian Li, Yangguang Lu

**Affiliations:** a Department of Nephrology, Wenling TCM Hospital Affiliated to Zhejiang Chinese Medical University (Wenling Hospital of Traditional Chinese Medicine), Taizhou, Zhejiang Province, China; b The First School of Medicine, School of Information and Engineering, Wenzhou Medical University, Wenzhou, Zhejiang Province, China.

**Keywords:** 17α-hydroxylase/17, 20-lyase deficiency, congenital adrenal hyperplasia, *CYP17A1* gene, diagnosis, genetic mutation

## Abstract

**Rationale:**

17α-hydroxylase/17,20 lyase deficiency (17OHD) is a rare autosomal recessive disorder that accounts for only 1% of all congenital adrenal hyperplasia. It has a high rate of misdiagnosis and mistreatment. However, the cases of repeated misdiagnosis and 3 times of mistreatment are extremely rare, which has important significance in medical education.

**Patient concerns:**

In this report, a 54-year-old female patient was transferred to the renal department due to proteinuria on physical examination. Enhanced adrenal computed tomography showed right adrenal hyperplasia with multiple nodules, likely adenoma. Blood tests showed low levels of renin, cortisol, estradiol, and androgens, and elevated levels of adrenocorticotropin. Uterine ultrasound indicated a rudimentary uterus. Genetic testing identified heterozygous variants of C.118A>T and C.316T>C in the *CYP17A1* gene.

**Diagnoses:**

After revising the diagnosis of primary amenorrhea, adrenal adenoma, and chronic nephritis, 17OHD was finally confirmed.

**Interventions:**

The patient received maintenance treatment with hydrocortisone (10 mg at 6 am and 10 mg at 2 pm).

**Outcomes:**

Postreatment, blood pressure, potassium levels, and urine protein normalized, with stable cortisol levels.

**Lessons:**

This case illustrates the importance of early and correct diagnosis of 17OHD through the patient’s tortuous medical experience, and all treatments should be very cautious before the accurate diagnosis of 17OHD. The possibility of 17OHD should be considered in patients with hypertension, hypokalemia, and insufficient puberty. When adult 17OHD patients cannot tolerate long-acting glucocorticoids, they can be replaced with hydrocortisone therapy.

## 1. Introduction

Congenital adrenal hyperplasia (CAH) refers to a group of rare autosomal recessive genetic disorders. CAH is classified into different subtypes based on the type of enzyme deficiency. 21-hydroxylase deficiency (21-OHD) is the most prevalent subtype, accounting for 95% of cases, with a global incidence of 1 in 10,000 to 1 in 16,000.^[1]^ In contrast, 17α-hydroxylase/17,20-lyase deficiency (17OHD) is much rarer, constituting only 1% of all CAH cases.^[2]^ Since its initial report in 1966,^[3]^ no precise incidence statistics have been established internationally. 17OHD primarily manifests as hypertension, hypokalemia, and disorders of sexual development.^[4]^ Although the diagnosis and treatment of 17OHD are generally straightforward, the condition has a notably high rate of missed diagnosis and misdiagnosis. Approximately 96% of cases are diagnosed during adolescence or later,^[5]^ and before the final diagnosis, approximately 94% of patients undergo various inaccurate diagnoses and treatments.^[6]^ This article presents a case of 17OHD that was misdiagnosed 3 times. This case is shared here to enhance clinicians’ awareness and understanding of the disease.

## 2. Case presentation

The patient is a 54-year-old female farmer who has never had children. She was admitted to the hospital due to persistent proteinuria for 1 year, along with symptoms of fatigue, chills, and nocturia occurring 3 to 4 times per night. She had a medical history of primary amenorrhea. During this period, she received artificial menstrual cycle treatment, after which menstruation successfully resumed. Additionally, she had a history of hypertension, breast implant surgery, and adrenal adenoma removal (histopathology report was unavailable). Her parents are not closely related, and she has an older brother who is in good health. She denied any history of smoking, alcohol consumption, or drug abuse.

First physical examination findings revealed a blood pressure of 150/96 mm Hg (1 mm Hg = 0.133 kPa), a pulse rate of 80 beats per minute, a height of 176 cm, a weight of 56 kg, and a body mass index of 18.1 kg/m². The patient had slender limbs and hyperpigmented skin, particularly on the face. No abnormalities were noted in the heart, lungs, chest, or abdomen. Genital examination was not performed. Second physical examination findings included the absence of axillary hair, breast implants in place, pale areolae, a juvenile-appearing vulva, a blind vagina, the absence of pubic hair, and no clitoral hypertrophy.

**Figure 1.**
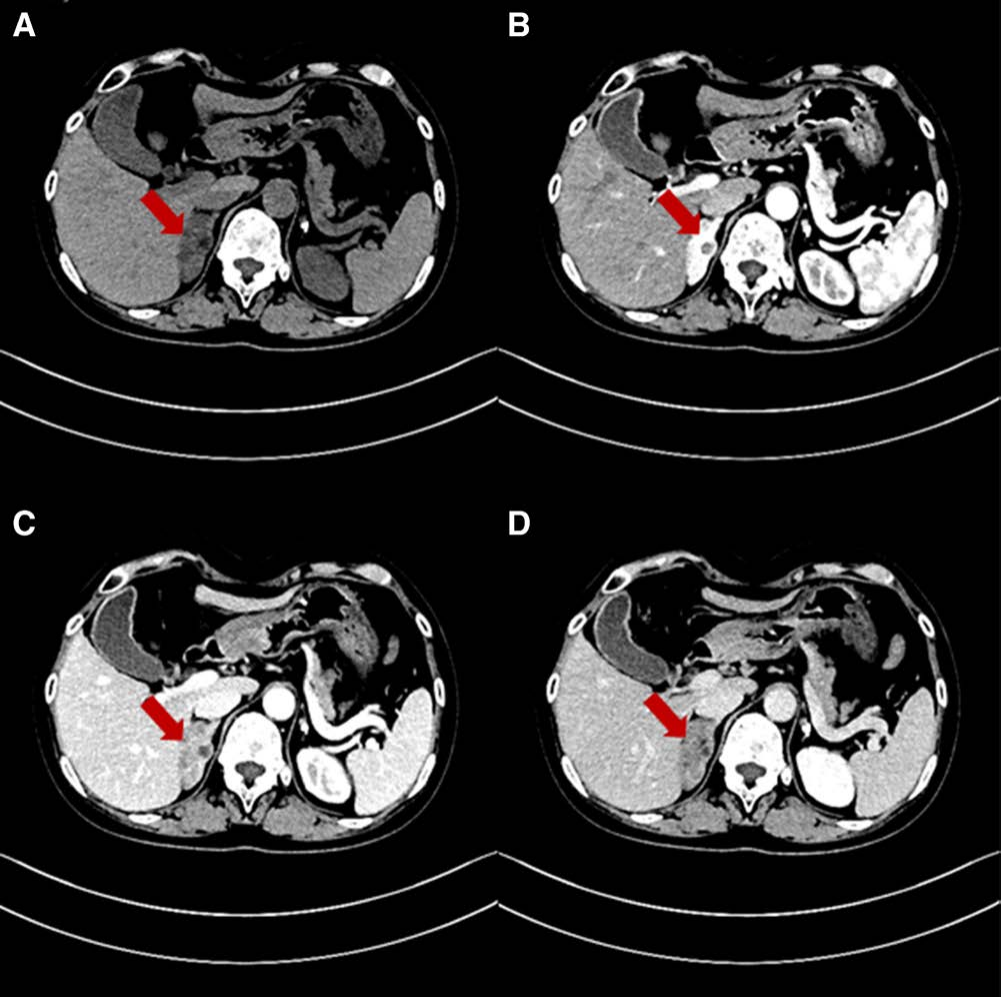
Adrenal enhanced CT results. (A) Normal sweep phase. (B) Arterial phase. (C) Venous phase. (D) Excretory phase. The red arrow indicates the enlarged adrenal gland. CT = computed tomography.

**Figure 2.**
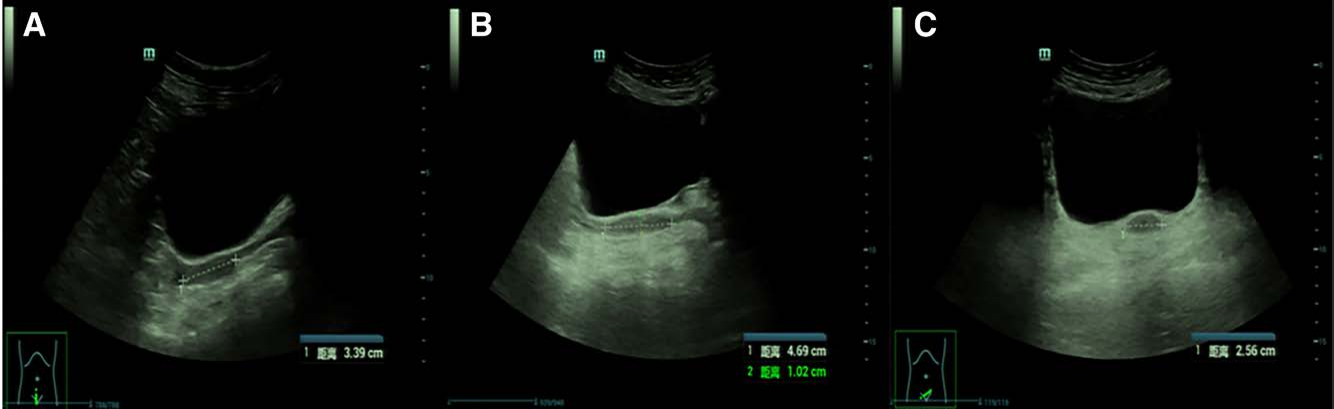
Uterine B-ultrasonography. The outline of the uterus is not clear; the size is about 34 × 10 × 26 mm. (A) An ultrasound image of the uterus with the measurement of the superoinferior diameter of the uterine body, which is 3.39 cm. (B) An ultrasound image showing multiple measurements. The distance from the uterine body to the cervix is measured as 4.69 cm, and the anteroposterior diameter of the uterine body is 1.02 cm. (C) An ultrasound image of the uterus with the measurement of the transverse diameter of the uterine body, which is 2.56 cm.

Based on initial laboratory results (Table 1), the patient was diagnosed with chronic nephritis, hypertension, and hypokalemia, and treated with valsartan 80 mg once daily. The patient’s urine protein, blood pressure, and potassium levels did not normalize. After incorporating amlodipine 5 mg once daily, blood pressure and proteinuria were controlled. However, blood potassium levels remained low. Given the adrenal abnormality observed on chest computed tomography (CT), an enhanced CT scan of the adrenal glands was performed, revealing hyperplasia of the right adrenal gland with multiple nodules and possible adenoma (Fig. 1). Subsequently, hormone level analyses (Table 2) showed low cortisol, estradiol, and androgen levels, elevated adrenocorticotropic hormone, and normal catecholamines, thereby excluding adrenal cortical adenoma and pheochromocytoma. After persuading the patient to undergo a second physical examination, abnormalities were found in the external genitalia and secondary sexual characteristics. Consequently, a uterine ultrasound was performed, revealing a rudimentary uterus (Fig. 2). Upon reviewing the patient’s medical history, we suspected congenital adrenal hyperplasia. Therefore, chromosome karyotype testing and genetic testing were conducted (Table 3). The results indicated that the patient was 46, XX and had a mutation in the *CYP17A1* gene. Based on these findings, we diagnosed the patient with 17OHD. Consequently, valsartan and amlodipine were discontinued, and treatment was switched to 0.75 mg dexamethasone tablets. Additionally, calcium carbonate D3 and rabeprazole capsules were prescribed as adjunctive therapy. After 16 days, the patient’s cortisol and adrenocorticotropic hormone levels normalized, and blood pressure, urine protein, and blood potassium also returned to normal. The patient reported improved skin texture but developed bilateral rib pain. A bone density test revealed osteoporosis, and alfacalcidol was added to the treatment regimen. However, the patient continued to experience discomfort. Considering potential intolerance to dexamethasone, the treatment was switched to hydrocortisone 10 mg at 6 am and 10 mg at 2 pm. After 1 year of irregular follow-up, the patient’s fatigue, chills, and nocturia improved. Blood pressure, potassium, and urine protein normalized, and cortisol levels remained stable.

**Table 1:** The results of the first laboratory examination.

Measure	Measured value	Reference range
Urinary protein	++	-
24-hour urine protein, mg/24 h	168.5	0.0–150
Serum potassium, mmol/L	3.01	3.5–5.5
Aldosterone(orthostatic), pg/mL	67.7	31.0–351.0
Renin(orthostatic), ng/mL/h	0.98	1.31–3.95
Angiotensin I (orthostatic), ng/mL	3.12	
Angiotensin II (orthostatic), pg/mL	53.91	32–90

**Table 2:** The results of various hormone tests.

Measure	Measured value	Reference range
F, ug/L	17.9	64–228 (8 am)
	8	25–100 (4 pm)
ACTH, pg/mL	95	7.0–65.0 (8 am)
	35.7	3.0–30 (4 pm)
PRL, ng/mL	7.47	1.2–29.93
FSH, mIU/mL	57.47	2.58–150.53
LH, mIU/mL	11.85	10.39–64.571
E_2_, pg/mL	<10	10–28
T, nmol/L	0.06	0.38–1.97
P, ng/mL	3.4	1.20–29.93
17a-OHP, nmol/L	0.15	≤6.11
CA, pg/mL	74.8	0–207

17a-OHP = 17a-hydroxyprogesterone, ACTH = adrenocorticotropic hormone, CA = catecholamine, E_2_ = estradiol, F = cortisol, FSH = follicle-stimulating hormone, LH = luteinizing hormone, P = progesterone, PRL = prolactin, T = testosterone.

**Table 3:** Mutations found in high-throughput sequencing.

Position	HGVS	Exon	MAF (East Asian)	Nature of mutation	ACMG prediction	ClinVar Classification
10: 104592289	c.1118A>T p.His373Leu	6	3/18384	Het	Likely pathogenic	Pathogenic
10:104595131	c.316T>C p.Ser106Pro	2	2/18394	Het	Pathogenic	Pathogenic

The gene detection report was obtained from Shanghai Endocrine and Metabolic Diseases Research and was detected by Ion_PGM platform. The human genome reference sequence version is human genome19 (hg19).

ACMG = American College of Medical Genetics and Genomics, HGVS = Human Genome Variation Society, MAF = minor allele frequency.

## 3. Discussion

17OHD is an autosomal recessive disorder caused by mutations in the *CYP17A1* gene. The *CYP17A1* gene, located at 10q24.3, consists of 8 exons and 7 introns and is expressed in the adrenal glands and gonads.^[7]^ The patient’s genetic test revealed heterozygous mutations, C.118A>T and C.316T>C, in the *CYP17A1* gene. These mutations are associated with 17OHD and render the patient genetically compound heterozygous. Due to the rarity of 17OHD, these mutations might be linked to the founder effect, particularly in regions with a historically small founding population or a relatively simple genetic structure. The founder effect can lead to certain genetic mutations occurring more frequently in specific populations compared to the general population. In regions with a small founding population or a history of genetic isolation, these mutations might occur more frequently. Clinical attention should focus on the potential impact of the founder effect, particularly in rare diseases like 17OHD. It is important to assess whether regional high-frequency genetic mutations occur, especially in East Asian populations.

The *CYP17A1* gene encodes cytochrome P450c17, which activates 17α-hydroxylase and 17,20-lyase. Deficiencies in 17α-hydroxylase and 17,20-lyase lead to impaired cortisol and sex hormone synthesis. Low cortisol levels activate the hypothalamus-pituitary-adrenal axis, causing an overproduction of adrenocorticotropic hormone (ACTH), thereby causing adrenal hyperplasia. Disruptions in sexual hormone synthesis affect both estrogen and androgen production. The patient’s karyotype was 46, XX, corresponding to a physiological female. Due to low estrogen levels, her sexual organs were immature. Estrogen reduction typically triggers the hypothalamus-pituitary-ovarian axis to increase follicle-stimulating hormone and luteinizing hormone secretion. However, this patient’s hormone levels remained normal, likely due to prior estrogen–progestin therapy and the degree of enzyme deficiency. Studies show that residual ovarian follicles in some 17OHD patients can cause elevated follicle-stimulating hormone and luteinizing hormone, leading to recurrent ovarian cysts. In complete 17OHD, no viable follicles are produced due to total enzyme loss.^[8]^ Therefore, this case represents a 46, XX patient with complete 17OHD. Androgen deficiency manifested as the absence of pubic and axillary hair, as well as bone metabolism abnormalities, consistent with the patient’s clinical findings. It is important to note that patients with 17OHD generally have a taller adult height due to delayed bone age, but short stature has also been reported.^[9]^ This is because height is regulated by multiple factors and should not be used as a definitive diagnostic criterion for 17OHD.

The inability to activate 17α-hydroxylase and 17,20-lyase also results in the accumulation of 17α-hydroxylase precursors, such as pregnenolone and progesterone, in the adrenal cortex. This leads to the synthesis of large amounts of deoxycorticosterone (DOC) through non-17α-hydroxylase pathways. DOC possesses 10%–20% glucocorticoid activity, which compensates for the deficiency in cortisol production, thereby preventing adrenal crises in most 17OHD patients.^[6]^ Additionally, DOC has strong mineralocorticoid effects, causing hypertension and hypokalemia in these patients. However, DOC is a precursor of aldosterone, and its overproduction leads to feedback inhibition of renin secretion, so the hypertension in 17OHD patients is actually a type of low-renin hypertension.^[10]^ The patient’s laboratory test results support this finding. Before diagnosing 17OHD, the patient was initially thought to have chronic nephritis with associated hypertension and hypokalemia and was treated with valsartan. However, as valsartan inhibits the renin-angiotensin-aldosterone system, it would further reduce renin levels, making it an inappropriate treatment for this patient. Therefore, clinicians, especially nephrologists, should rule out 17OHD before prescribing renin-angiotensin-aldosterone system inhibitors. The patient had experienced 2 previous misdiagnoses. The first misdiagnosis occurred during adolescence when the patient was diagnosed with primary amenorrhea, but the underlying cause was never identified. Studies in Turkey have shown that approximately 25% of 17OHD cases are diagnosed before the age of 10 years, with delayed puberty as the most common complaint.^[11]^ However, this patient was not diagnosed until she was 55 years old, which could be attributed to the medical standards of the time and the patient’s social stigma. Studies have shown that 46 XX patients can achieve pregnancy through sequential estrogen-progesterone therapy and assisted reproductive techniques.^[12]^ Unfortunately, this patient missed the optimal treatment window. The second misdiagnosis occurred 10 years ago, when the patient was diagnosed with an adrenal adenoma and underwent surgical resection. In reported cases of 17OHD combined with adrenal masses, many patients have undergone adrenal mass removal. In addition to adrenal hyperplasia, postoperative pathology has shown adrenal cortical adenomas^[13]^ and adrenal myelolipomas.^[14]^ However, in patients with 17OHD who did not undergo surgery, follow-up revealed that their adrenal masses gradually shrank after dexamethasone treatment, indicating that high levels of adrenocorticotropic hormone likely contribute to secondary tumor formation on the basis of adrenal cortical hyperplasia.^[15]^ Therefore, clinicians should carefully consider adrenal mass surgery only after confirming a 17OHD diagnosis. In addition, if adrenal-related diseases are clinically suspected, it is recommended to proceed with CT imaging for further evaluation, even if ultrasound findings are normal.

17OHD is a congenital disorder. Based on the current medical level, the disease remains incurable, and treatment focuses on managing symptoms. Treatment goals for 17OHD vary by age, but glucocorticoids are the cornerstone of therapy across all age groups. Exogenous glucocorticoids replace deficient cortisol, restoring negative feedback to the hypothalamus-pituitary-adrenal axis and reducing adrenocorticotropic hormone secretion, thereby inhibiting adrenal hyperplasia. Long-acting glucocorticoids are preferred for adult 17OHD patients. Initially, the patient was treated with dexamethasone, but due to intolerance, it was replaced with hydrocortisone. Clinical indicators showed that hydrocortisone therapy remained effective. Additionally, treatment for 17OHD includes gender selection and complication management. This patient identifies as female both physically and psychologically, so gender reassignment is unnecessary. Long-term hypertension in 17OHD patients often leads to target organ damage, most commonly affecting the kidneys and eyes,^[16]^ which explains why this patient had increased proteinuria and nocturia. After glucocorticoid replacement therapy, the patient’s blood pressure was controlled. Therefore, no additional antihypertensive drugs were needed. However, long-term glucocorticoid therapy may worsen osteoporosis symptoms in 17OHD patients and increase the risk of steroid-induced diabetes. Thus, for these patients, long-term follow-up is essential, and glucocorticoids should be maintained at the lowest effective dose.

## 4. Conclusions

We report a case of 17OHD that was misdiagnosed 3 times. Accurate diagnosis requires a thorough analysis of clinical manifestations, biochemical tests, imaging studies, and genetic testing. Early identification and intervention are critical to improving patient outcomes.

## Acknowledgments

All authors would like to express their gratitude to the patient mentioned in this paper for his permission to allow us to report on this case.

## Author contributions

**Conceptualization:** Jian Li.

**Funding acquisition:** Jian Li.

**Methodology:** Jian Li.

**Resources:** Jian Li.

**Visualization:** Jian Li.

**Writing – original draft:** Jian Li.

**Writing – review & editing:** Jian Li, Yangguang Lu.

**Supervision:** Yangguang Lu.
